# Environmental health and justice and the right to research: institutional review board denials of community-based chemical biomonitoring of breast milk

**DOI:** 10.1186/s12940-015-0076-x

**Published:** 2015-11-25

**Authors:** Dvera I. Saxton, Phil Brown, Samarys Seguinot-Medina, Lorraine Eckstein, David O. Carpenter, Pamela Miller, Vi Waghiyi

**Affiliations:** Department of Anthropology, College of Social Sciences, California State University, Fresno, 5242N. Backer Ave. Peters Business Building M/S 20, Fresno, CA 93740 USA; Northeastern University, Social Science Environmental Health Research Institute, 318 INV, Boston, MA 02115 USA; Alaska Community Action on Toxics, 505W. Northern Lights; Suite 205, Anchorage, AK 99503 USA; University at Albany, Institute for Health and the Environment, 5 University Pl., Rm. A217, Rensselaer, NY 12144 USA; Native Village of Savoonga Tribal Member, St. Lawrence Island, AK USA

**Keywords:** Biomonitoring, IRBs, Breastmilk, Alaska Natives, Right-to-know, Environmental justice, PCBs, Organochlorine pesticides

## Abstract

Recently, conflicts and challenges have emerged regarding environmental justice and research ethics for some indigenous communities. Alaska Community Action on Toxics (ACAT) responded to community requests for breast milk biomonitoring and conceived the Breast Milk Pilot Study (BMPS). Despite having community support and federal and private funding, the BMPS remains incomplete due to repeated disapprovals by the Alaska Area IRB (Institutional Review Board). In this commentary, we explore the consequences of years of IRB denials, in terms of health inequalities, environmental justice, and research ethics. We highlight the greater significance of this story with respect to research in Alaska Native communities, biomonitoring, and global toxics regulation. We offer suggestions to community-based researchers conducting biomonitoring projects on how to engage with IRBs in order to cultivate reflective, context-based research ethics that better consider the needs and concerns of communities.

## Background

This commentary examines some of the long-term consequences following an Institutional Review Board (IRB) rejection of a community-based participatory research (CBPR) project. Recently, Alaska Native communities collaborating with Alaska Community Action on Toxics (ACAT) have faced challenges from an IRB. ACAT is an environmental health, justice, research and advocacy organization that works with Alaska Native communities to address concerns about environmental contaminants and community health. In 2005 in response to community requests for biomonitoring research, ACAT, partnering tribes and academic researchers initiated the Breast Milk Pilot Study (BMPS). The project sought to measure levels of persistent chemicals in breast milk, promote breastfeeding, address concerns about toxic exposure and health, and support women’s right to know about toxics in their bodies. These goals are supported in the scientific literature [1, 2], however the BMPS remains incomplete.

Although the Belmont Report and other sources stress the importance of protecting human subjects from harm and of emphasizing research benefits, some IRBs may have difficulty in reaching a balance between protecting individual human subjects and facilitating research of crucial importance to the subjects and the communities they are attempting to protect. In this commentary, we address the challenges ACAT faced following repeated disapproval of their project by the Alaska Area Institutional Review Board (AAIRB). We explore the short and long-term consequences, examining outcomes with respect to structural health inequalities and research ethics. We highlight the greater significance of this story regarding research with global indigenous communities who are often exposed to environmental pollution and its effects on health and cultural survival. We pose and reflect on the following questions: (1) what are the ethical ramifications when carefully planned research inspired by and developed in collaboration with indigenous communities is repeatedly denied IRB approval? What does it mean when assumptions about the inherent vulnerability [3] of indigenous peoples prevent them from exploring environmental health problems that may be exacerbating health disparities in Native communities?

This commentary is based on a thorough review of ACAT’s archives and documents covering the AAIRB process and ACAT’s efforts to develop, fund, and carry out the BMPS in collaboration with academic and Alaska Native community partners. We conducted interviews with a former IRB official, researchers, and clinicians who have worked with ACAT and who have long-established relationships with Alaska Native communities. We engaged in critical self-reflection with ACAT staff (Seguinot-Medina, Miller, Waghiyi, Eckstein) and an affiliated researcher (Carpenter) about conflicts with the AAIRB that affected morale and the communities’ goals to use research findings to support toxics regulation and local cleanup efforts. Researchers at Northeastern University (Saxton and Brown) provided outside analysis of ACAT staff and researchers’ insights and conducted an extensive literature review about breastfeeding and biomonitoring research in which researchers report results to research participants. This commentary project was approved by Northeastern University’s IRB.

### Indigenous Research, IRBs, and Ethical Nuance vs. Imperialism

For indigenous people throughout the world, research, in and of itself, “is not [necessarily] considered a societal good” [3]. Some indigenous people in the U.S. share this sentiment, given the long history of the exploitation and the dismissal, devaluation, appropriation, and desecration of their beliefs, practices, knowledge, environments, bodies, and bodily substances [4]. However, there are sincere efforts to do research that validate indigenous knowledge and prioritize their concerns. Community-based participatory research (CBPR) is a partnership approach that equitably involves community members in the conceptualization, design, implementation, and evaluation of research projects Certainly, these efforts are not without challenges, but productive relationships have been sustained in environmental health research projects with Native Americans and Alaska Natives [5].

By U.S. law, research with American Indians and Alaska Natives requires intensive reviews by multiple IRB committees. In the U.S., the Indian Health Service (IHS) under the Department of Health and Human Services charges Area IRBs with evaluating research projects involving American Indians and Alaska Natives. Area IRBs are supposed to include volunteers from diverse backgrounds: Native and non-Native, researchers, clinicians, and community leaders. Some tribes also have internal IRBs that serve to vet proposed projects conducted by the tribe itself. Regional Indian Health Centers and clinics (IHCs) also evaluate projects involving biomedical and public health research and interventions. In Alaska, there are nine IHCs, including the NSHC. The approval of the indigenous group and IHC is required before an Area IRB will review a proposal. If researchers are affiliated with another institution, such as a university or a hospital, those IRBs must also approve the project.

Still, in some cases, IRBs exert “ethical imperialism” [6], wherein universal ethical frameworks are used to evaluate projects irrespective of the expectations that indigenous people hold about their health and environment. In other cases, the values, political beliefs, and liability concerns of institutions (e.g. universities, hospitals, state agencies) and the IRBs that serve them can shape whether or not a proposal gets approved [7]. There is also a tendency to “homogenize” indigenous people as inherently vulnerable “regardless of their particular social position” [3], their relationships to researchers, or their intention to consent.

Certainly, our intent in writing this commentary is not to undermine the efforts of those IRBs that are sincerely concerned with potential negative consequences by researchers. It is important to have people from diverse academic and community backgrounds on IRBs. It is also important that indigenous persons vet projects and ensure they are in line with the priorities of the participating indigenous communities as well as any applicable legal and cultural standards [8]. IRBs can help foster important discussions about the ethical implications of research. They can also help researchers, whose values are sometimes clouded by their own professional ambitions or lab-bench visions, to improve their cultural competency and real-world ethical nuances. The IRB approval process can involve a cooperative process of dialogue, feedback and revisions to ensure that researchers have reflected upon and incorporated ethical concerns into their research designs. These discussions can also help IRB members understand different approaches to research. However, this was not the case for the Alaska Area IRB (AAIRB) when it reviewed the BMPS. As of this writing (November 2015), the BMPS remains unapproved and unresolved.

### ACAT and the Breast Milk Pilot Study (BMPS)

**Fig. 1:**
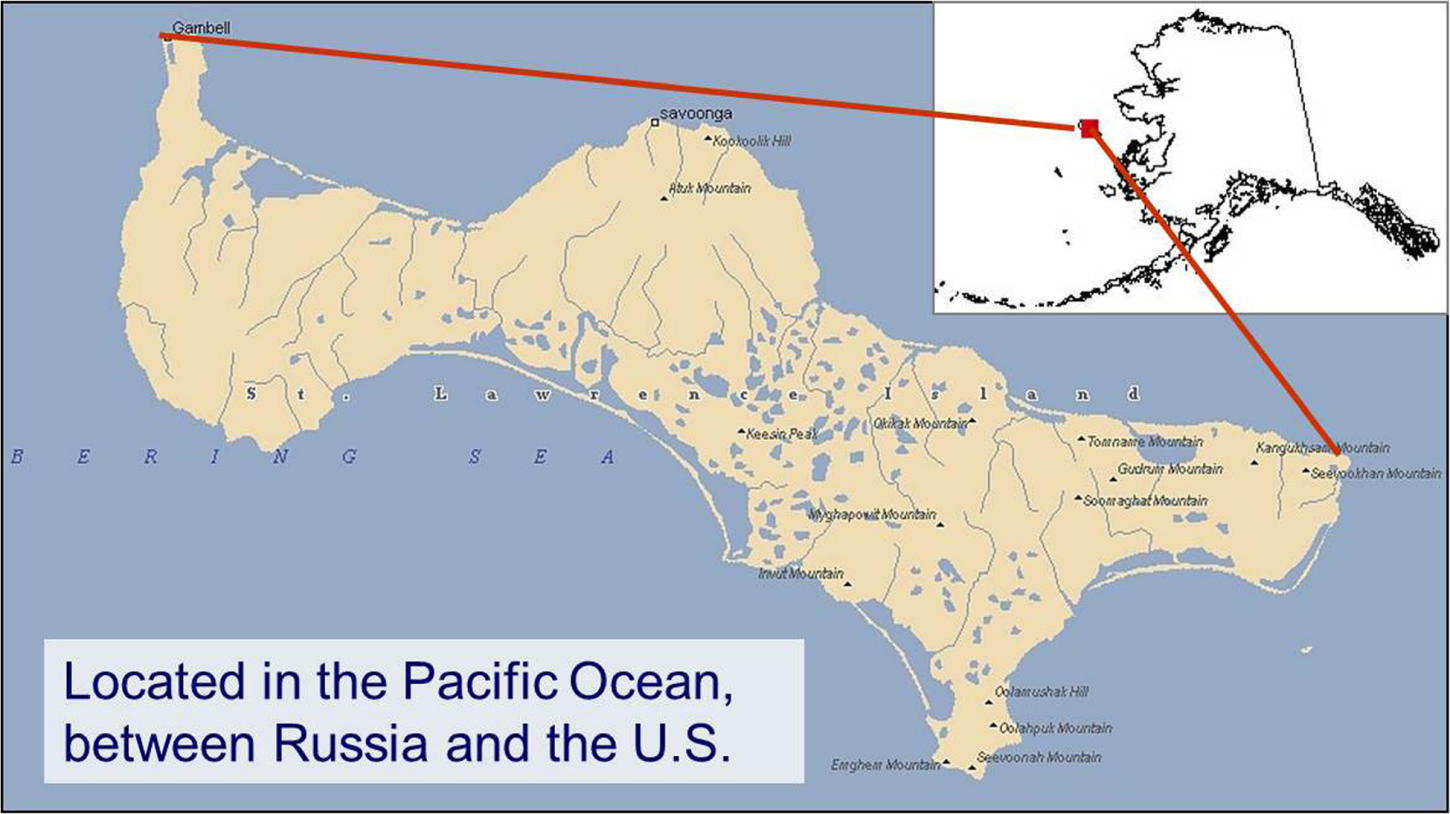
Map of Saint Lawrence Island

For eighteen years, ACAT has built long-standing, culturally and ethically grounded relationships with Alaska Natives, especially with the Yupik people on St. Lawrence Island: located in the Northern Bering Sea (Fig. 1). Annie Alowa, a Yupik elder, helped establish ACAT following decades of observing trends of cancers, low birth weights, and miscarriages in her community. ACAT’s board and research team include nine Alaska Natives. Together they have led efforts to research potential environmental causes of diseases and to demand state and military accountability to clean up toxic military dump sites on St. Lawrence Island. They also prompt effective regulations and bans at the state, national, and international levels on toxic chemicals produced far away from the Arctic but found in the bodies, subsistence foods, and environments of St. Lawrence Island Yupik and other Arctic peoples.

The BMPS was conceived in 2005 following a study that analyzed PCB levels in the blood of the St. Lawrence Island Yupik People [9]. ACAT partnered with environmental health scientists at the State University of New York at Albany (SUNY Albany) to conduct the BMPS. Together, they prepared proposals for potential community collaborators, funders, the Norton Sound Health Consortium (NSHC—the primary health care provider for Alaska Natives in the Norton Sound region), and the AAIRB. ACAT received letters of support from tribal councils and community organizations including the Native Villages of Savoonga and Gambell on Saint Lawrence Island, and the Native Villages of Brevig Mission, Diomede, White Mountain, and Unalakleet. It featured questions and hypotheses posed by St. Lawrence Island Yupik people and a culturally sensitive and scientifically appropriate methodology for conducting research on breast milk contaminants. Kawerak, Inc., a tribal non-profit organization and the NSHC were also supportive.

**Fig. 2:**
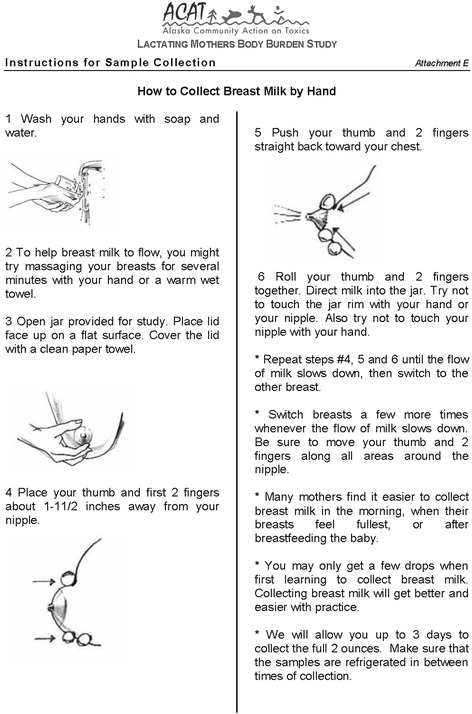
ACAT’s breast milk self expression instructions

**Fig. 3:**
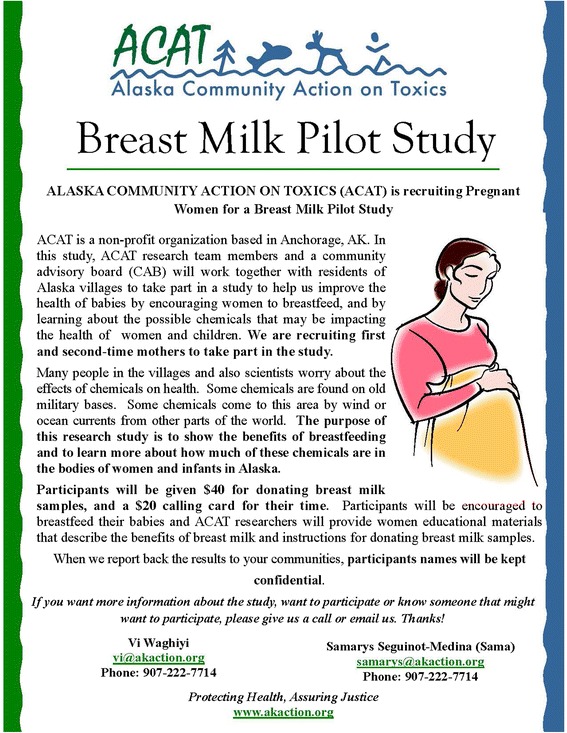
ACAT BMPS recruitment flyer

On January 5, 2005, ACAT and research partners from SUNY Albany prepared a grant application to the NIEHS to fund an environmental justice CBPR project that included environmental monitoring in the Norton Sound region as well as the BMPS. The BMPS included a non-invasive breast milk sampling protocol. Each participant would receive a special kit with a manual breast pump, instructions on how to collect samples (Fig. 2), and carefully designed materials that encouraged sustained breastfeeding (Fig. 3). Participants would self-express 10 ml of milk at home at one month and three months postpartum. Samples would be analyzed for two persistent organic pollutants (POPs), organochlorine (OC) pesticides and congeners of PCBs, including those found at formerly used defense sites (FUDS) in the region. In addition, one venous blood draw (<50 ml) would be taken from pregnant participants at a routine prenatal checkup in their eighth month of pregnancy to identify health markers (such as thyroid hormone levels) that might correlate with the levels of contaminants in breast milk.

To quell community members’ potential concerns about infants consuming tainted breast milk, the BMPS included a breastfeeding intervention. This included a lactation consultation and culturally appropriate breastfeeding educational materials detailing the many documented social, cultural, economic, and health benefits of breastfeeding. The materials also referenced literature about the role of breast milk in potentially reversing harms induced by exposure to contaminants in utero [10].

Upon receiving the NIEHS grant, on March 8, 2007 ACAT submitted an application to the AAIRB. Two months later, on May 25, 2007, the AAIRB sent written notice to ACAT and researchers requesting: (1) results from similar research studies conducted on St. Lawrence Island, (2) more details on ACAT’s relationships with the Yupik and researchers from SUNY Albany, (3) additional discussion of the risks and benefits, (4) clarification of the purpose and research methods, (5) rationale for using blood to test for thyroid hormones, and the removal of this test as a listed benefit to participants (many of whom lack regular access to health care providers given their geographic isolation), (6) justification of the small sample size, (7) more information on the report-back component, (8) description of the disposition and labeling of the specimens, and (9) changes to the consent form and its language. ACAT responded on June 15, 2007, making the requested changes and clarifying the project’s proposed methods and activities.

On October 21, 2007, the AAIRB responded to ACAT’s revisions and clarifications, indicating that they consulted with an outside expert in their review. The AAIRB stated that the BMPS was of “limited scientific value” and expressed concerns that reporting back breast milk contaminant levels would discourage Native mothers and others from breastfeeding and from eating potentially contaminated foods.

In 2008, ACAT prepared and submitted a second application to the AAIRB, paying careful attention to concerns about breastfeeding cessation and reporting back of results. ACAT’s Research Anthropologist Lorraine Eckstein (one of the authors) has considerable expertise with human subjects requirements, including: two years serving on a biomedical IRB and one year on a sociological IRB at the University of Washington, and eight additional years at ACAT serving as the Human Protections Administrator for Federalwide Assurance (FWA) for the Protection of Human Subjects. Eckstein assured that researchers on ACAT’s research team complied with IRB regulations for the protection of human subjects including biomonitoring research with Alaska Natives. The Native Village of Savoonga and the tribal non-profit organization Kawerak, Inc. contributed letters of support. ACAT presented the project to the NSHC Ethics Review Board (NSHC RERB) to explain the study further. In 2009, the NSHC RERB indicated for the second time that it would approve the project pending the AAIRB’s approval. ACAT submitted the application to the AAIRB, but never received a response.

A few years later, in a meeting at the ACAT office (March 9, 2011), the AAIRB Administrator offered a verbal apology on behalf of the AAIRB. The Administrator explained that the AAIRB was changing their paper application process to the IRBNet electronic submission system, and that it was possible that ACAT’s application might have gotten lost in that process.

On January 5, 2010, ACAT received a Passport Foundation Grant to supplement additional analyses for the BMPS. In its third application to the AAIRB, the ACAT research team emphasized how the study would *encourage* breastfeeding with educational materials, and cited science on breast milk’s health protective properties. Once again, participant information packets included this information along with the sampling kits and instructions. The protocol explained that breast milk and blood samples from “approximately 40 lactating mothers in Arctic Alaska communities” would be analyzed in a lab for various contaminants. This application also justified the small sample size as appropriate for a pilot study.

Having worked with SUNY Albany before, ACAT thought it could move the BMPS forward through their university IRB, which approved the project on May 17, 2010. Due to the obligations to the tribes that requested this study, the research team commenced with participant recruitment. However, on January 29, 2011, a letter from NSHC called for the immediate suspension of research, pending AAIRB approval. ACAT and researchers from SUNY Albany stopped recruitment and prepared a third application to the AAIRB.

Although the third application (January 2010) to the AAIRB incorporated suggestions and feedback from NSHC RERB, the AAIRB, and outside advisers, the AAIRB sent ACAT a letter dated June 8, 2011 requesting that changes be made to the BMPS screening process and that the ACAT list benefits and risks. The AAIRB recommended that ACAT consult with an outside expert about using biological samples other than breast milk as proxies.

One of the authors, David O. Carpenter, Director of the Institute for Health and the Environment at SUNY Albany designed the sampling methodology for the BMPS. He explained that while the blood levels and the breast milk levels are totally interrelated, the importance of testing breast milk is that, unlike blood, breast milk is transmitted directly from mothers to infants and children. Over time, breastfeeding flushes chemicals out of a woman’s body and into her child’s body. Thus, a breast milk sampling mechanism would help scientists determine children’s exposures throughout their breastfeeding time. Breast milk, as Smolders et al. [11] observe: “is a major uptake route for environmental contaminants […] and represents the main exposure source for breast feeding infants.” During an interview with Saxton, Carpenter suggested that another reason for testing breast milk is that researchers “will get the same information [regarding kinds of contaminants] from blood, [but] *the concentrations* aren’t going to be as high … [The tests] are much more sensitive if you use breast milk because the fat content is high.” Results can also be compared to global monitoring of breast milk conducted by the World Health Organization and others.

ACAT responded to the AAIRB on June 27, 2011 and complied with the requests, but kept the breast milk biomonitoring component to honor the original Alaska Native-driven research questions. They attached six letters of support from Alaska Native communities. The leaders of the Native Village of Savoonga on St. Lawrence Island detailed why they wanted to do the BMPS:

We believe that this project is a good way to increase our knowledge about toxics on our Island, our foods, and our Yupik people. We…hope that the results will be helpful for us to make changes to environmental laws and policies that will protect the health of our children and our future generations. [unpublished observation June 2011].

Another supporting community expressed concern about contaminants, traditional foods, and children’s health:

We depend on our ocean for food and are concerned about the sick seals, walrus, and polar bear. We need to know if we are consuming contaminants from the food…and passing it on to our babies we breast feed. [unpublished observation June 2011].

Armed with such support, in their IRB application cover letter, ACAT requested a meeting with the AAIRB to discuss additional questions or concerns. On September 30, 2011, ACAT received a denial letter from the AAIRB.

On January 5, 2012, ACAT team members visited the AAIRB office to schedule a meeting. An AAIRB committee member agreed to meet and discuss concerns and strategies to get the BMPS approved. ACAT also sought advice from staff at NSHC. On February 21, 2012 ACAT leaders and team members gave a 10-minute presentation to the AAIRB, during which committee members had opportunities to make comments, ask questions, or voice concerns. None were expressed, and the AAIRB administrator indicated that they would review the presentation and application and contact ACAT with any additional questions or comments.

On May 18, 2012, after five years of failed communications, three denied applications, an expired NIEHS grant, and an extension from the Passport Foundation, ACAT sought legal counsel from a local law firm to discuss the BMPS and potential legal actions that could help facilitate the approval and completion of the project. On May 25, 2012 ACAT submitted a new application to the AAIRB.

On July 11, 2012, ACAT received a modification request letter from the AAIRB asking for clarification of the specific study sites in the protocol. The letter also stated: “Researchers must form a Community Advisory Board (CAB) from each community represented. The IRB strongly recommends including NSHC nursing moms as CAB members and/or a lactation consultant from the region.” The AAIRB requested an official CAB roster, prohibited members of the BMPS research team from being on the CAB (including Alaska Native researchers with decades of experience living and working in the communities), recommended that nursing moms and/or a lactation consultant from the region be included as CAB members, and solicited complete documentation of CAB meetings, including dates, agendas, and minutes. ACAT was willing to form a CAB and provide minutes; however, the suggestion to form a CAB from each participating community was impractical for this pilot study, which was designed to have a small number of participants from each community. The AAIRB also recommended that a NSHC health care provider, rather than the SUNY Albany research partner, conduct the report-back results. The AAIRB also required more revisions to the consent form and recruitment flyer, new letters of support from each participating community, and official approval from the NSHC Board of Directors (which would not give approval unless the AAIRB approved the study first).

On August 15, 2012 ACAT submitted a letter to the AAIRB asking for clarification on this latest modification request. An AAIRB administrator responded to the concern about the CAB in an email: “Demonstrate that the CAB has discussed this project and the potential negative consequences to *ALL* breastfeeding mothers and how this can be mitigated.” The AAIRB’s concern that the dissemination of the study findings would discourage breastfeeding by mothers throughout Alaska continued, leading the AAIRB to request the use of blood and tissue instead of breast milk:

We have consulted a recognized expert on contaminants and after much discussion [the committee] has determined that the IRB would approve this project if the investigators considered collecting blood instead of breast milk. . . . The collection of blood would a) eliminate the potential risk of decreasing breastfeeding, b) allow for testing of additional contaminants that are not found in breast milk, and c) blood is collected on a routine basis and could be more acceptable. [Unpublished observation August 2012]

The identity of the outside experts referenced in the AARIB’s letters was never shared with ACAT, despite numerous requests for this information.

In a final effort to move the BMPS forward, leaders from the St. Lawrence Island Native Villages of Gambell and Savoonga sent letters to the AAIRB, expressing their concerns about the health issues their communities are facing, thanking them for approving past projects, and urging them to reconsider their decision. On October 12, 2012 ACAT submitted a letter and supporting documents through their attorney, seeking answers and clarification on why the BMPS had never been approved after more than five years of concerted effort to comply with all of the AAIRB’s extensive and repetitive requests, requirements, meetings, presentations, protocol revisions, consultations with experts, and modifications. Ultimately, ACAT decided to halt the BMPS due to exhaustive modifications required by the AAIRB and extenuating staff, resource and financial circumstances related to sustaining the project.

### 10 years later: responding to the AAIRB

Here we analyze challenges that shaped ACAT’s interactions with the AAIRB. Specifically, we use scientific literature on breast milk biomonitoring and contaminants, including case studies of previous community-based participatory and biomonitoring research and indigenous feedback to critique the AAIRB’s: (1) fears about contaminants in breast milk and traditional foods, (2) reservations about results report-back, and (3) differing ideas about using research to support advocacy efforts. We pose new questions about the roles that IRBs play and discuss the potential consequences that ethical imperialism can have for indigenous communities wanting to conduct their own research on environmental health disparities.

#### Fears about contaminants in breast milk and traditional foods

The BMPS project sought to study the presence of 101 POPs congeners, including PCBs and 28 organochlorine (OC) pesticides, some of which were banned decades ago (e.g. DDT) but still persist in the land, soil, water, and subsistence foods of the Arctic, as well as the bodies and breast milk of Alaska Natives. Since POPs build up in body fat, there are three primary matrices for assessing the body burdens of these chemicals: breast milk, blood, and fatty tissue. While maternal and fetal exposure may be evaluated through blood serum and cord blood analyses, breast milk sampling facilitates understanding exposures of the breast-fed child [12–14]. POPs bioaccumulate in fat; thus, wildlife and people at the top of the food chain have the highest risk of exposure. With breast-fed infants at the very peak of the food chain, it is even more critical to assess nursing mothers’ exposure to POPs through breast milk biomonitoring [13].

Breast milk is a relatively easy and non-invasive matrix to collect [11, 13, 15, 16]. It gives participants control over the sampling process, enabling them to collect the sample in the privacy of their own homes (vs. having a phlebotomist do it for them, as in the case of blood). Still, lactation doesn’t come easily to all new mothers [17], and post-partum tiredness and fatigue can make participation in research projects challenging [18]. Many of these concerns can be addressed through study and recruitment design and by incorporating participant feedback into projects in real time, especially via CBPR approaches [13, 19–21].

With respect to the AAIRB’s claims that the dissemination of the findings from the BMPS would discourage breastfeeding amongst Alaska Native mothers, there are multiple factors, some far more structurally and socially embedded than fear of environmental contaminants, that inhibit breastfeeding. In the U.S. context, breasts, breastfeeding, and breast milk are highly politicized and emotive [13], and breastfeeding as a practice receives substantially less social and political support than in Europe and other parts of the world. Records from the 1950s show that health workers encouraged Alaska Native mothers to use formula and discouraged the consumption of traditional foods [22–24]. Now, health care providers, scientists, and Alaska Native community leaders are working to re-validate Arctic indigenous health and food practices and knowledge, including support for breastfeeding. In the past, it was not uncommon for Alaska Native mothers to breastfeed into the third and fourth years of a child’s life [23].

The goals of improving breastfeeding rates and understandings of environmental health hazards need not be mutually exclusive or contradictory [1, 13, 25–27]. Indeed, the World Health Organization leads breastfeeding promotion efforts and hosts a global breast milk biomonitoring program [28]. As Boswell-Penc urges:

Unless we come together, locally as a nation, and as a global collective—and begin addressing the complex set of issues that add up to practices that pollute the environment […] women will no longer have the choice to nurse their babies if they want to protect them from the toxins that will have accumulated in the fatty tissues of their bodies [25].

Still, the AAIRB repeatedly asserted concerns that the BMPS would discourage breastfeeding not only in participating communities, but also in Alaska Native and non-Native communities that were not participating in the project. They also suggested that the project would discourage people from eating traditional subsistence foods. They did not provide evidence to back up these claims. By advising ACAT to switch from breast milk to blood biomonitoring, the AAIRB foreclosed opportunities for community research-based advocacy that could have long-term consequences for environmental health and justice.

The AAIRB, in their assessment of the BMPS, did not consider the potential benefits of community right-to-know and the role that the BMPS could have played in empowering Alaska Native communities to make their own informed decisions. The Yupik people on St. Lawrence Island and ACAT have learned from previous CBPR projects that dietary changes alone will not stop the contamination of their environment, their bodies, and their traditional foods. Native communities view contamination of their food and their bodies as human rights violations that will cause harm and social suffering for present and future generations. They work to mitigate and prevent those harms by participating in individual-level interventions, demanding that contaminated sites be cleaned up and remediated, and by urging for policy changes at the local and global levels. Having environmental health data to support these efforts is critical to ensuring that people will have both the right and the choice [25] to eat traditional foods and breast feed well into the future.

#### Reservations about report back

LaKind et al. [29], WHO [14] and others have assessed the strengths and weaknesses of various study designs and report-back methods in breast milk biomonitoring projects. Communities that participate in biomonitoring and environmental health research when results are reported back can benefit from learning their personal and collective results. The project identified as MOMs and POPs (Making Our Milk Safe or MaPP) is an example of a breast milk biomonitoring project that encourages breastfeeding alongside community right to know. Results are used to inform individuals about their toxic body burdens, to empower people to make changes that will reduce or eliminate potential risks, and to mobilize the data to strengthen international toxics regulations [13, 20, 21, 30, 31].

In addition to pro-breast feeding materials, the BMPS included a thorough post-project evaluation survey. Participants were to be interviewed about their experiences with the recruitment procedures, the breastfeeding guides and support, and the materials that explained breast milk sampling procedures. The survey also would have explored whether or not the mothers wished to receive their biomonitoring results and how the information affected them. Dr. Carpenter had planned to provide private consultations with each participant, as he had done for past biomonitoring projects on St. Lawrence Island [9]. The report backs would have included a detailed and careful overview of the results and a discussion of known, possible, and unknown health effects. The project would have strongly encouraged all participants to continue breastfeeding, reiterating research indicating that greater exposures to environmental contaminants take place in utero, and that the beneficial properties of breast milk may help mitigate the health impacts of prenatal exposures [13, 32].

The BMPS was inspired in part by the women of Akwesasne, a Mohawk community located near the heavily industrially contaminated St. Lawrence River in Upstate New York. Mohawk women requested a breast milk biomonitoring study in the late 1980s, and the New York Department of Public Health worked with the tribes to design and implement the project [33]. Katsi Cook, a Mohawk midwife and community-based researcher, observed a resurgence of traditional indigenous birthing and healing practices, including breast feeding. Mohawk women came to these decisions on their own terms after weighing the evidence [34]. The findings brought attention to the long legacies of dumping and contamination on Indian lands, helped people make decisions about their health, and supported demands for the cleanup of contaminated sites [25-35, 36].

It would be more appropriate to hypothesize Alaska Native women’s responses to their breast milk biomonitoring results based upon the Mohawk experience at Akwesasne or those that guide the First Nations Biomonitoring Initiative [37]. Instead, AAIRB officials and committee members relied on their own unsubstantiated fears with respect to mothers’ responses to breast milk biomonitoring results, and a paternalistic attitude about how Alaska Natives make health decisions. There is enormous potential for carefully designed biomonitoring studies with conscientious and clear report back protocols to have positive effects at the individual, clinical, community, and public health policy levels [20, 38–40]. This body of evidence counters the AAIRB’s fears of widespread anxiety and decreased breastfeeding and traditional foods consumption. Their claims also devalue the efforts of St. Lawrence Island Yupik people to collaborate with environmental justice organizations and scientists to validate their own hypotheses about contaminants and to make their own health decisions. The St. Lawrence Island Yupik see this as an affront to their communities’ efforts to raise healthy children and to ensure the cultural and biological survival of future generations.

More broadly, in considering the underlying assumptions of the study and its rejection by the IRB, we note the central issues of risks vs. benefits of reporting results of breast milk biomonitoring. There are several considerations here. The first issue is the question of whether it is ethical to not report back results of any biomonitoring. In our judgment it is not. If biomonitoring is done, research participants have a right to know the results and to have them explained in an understandable fashion. The only possible risk would be that a woman might decide that it was unwise for her to continue to breastfeed when that was not the case. Under certain circumstances it may be unwise for a women to breastfeed. When certain chemicals that are known to cause cognitive defects in children are found in breast milk at sufficiently high concentrations, it may be unwise for the mother to continue to breast feed, but this is unlikely. In the first place the infant’s greatest exposure to the mother’s body burden of contaminants is prior to birth, the period of time when brain and other organ systems are developing. This exposure has already occurred well before breastfeeding commences. Secondly, the benefits of breastfeeding on cognitive and immune function are extremely well documented. While breast feeding does increase the chemical body burden of the infant, almost all of the evidence to date indicates that post-natal exposure has much less effect on cognitive development as compared to prenatal exposure.

There are significant benefits to reporting results back to women who donate breast milk to a CBPR study. The chemicals in breast milk reflect the maternal exposure via diet and other pathways, and the knowledge of what is in her breast milk can be a basis for her making decisions about diet and other routes of exposure. This will be a health benefit to the mother, the breastfeeding child, and any future children, as information about contaminants in breast milk can influence greater community patterns of diet and activity. This is above and beyond the benefits to the greater scientific community in learning patterns of exposure which then can lead to study of the associations, if any, between exposure and disease. The benefit of overall knowledge of contaminant levels in breast milk is more to the next generation and to the community, should mothers and other community members choose to change dietary and other sources of exposure.

Results must be explained to individual women participants by someone who is culturally competent, knowledgeable of the known benefits of breastfeeding as well as the known risks of exposure to lipophilic chemicals. There is always a risk from inaccurate or biased communication; however, we do not see circumstances where the risks of reporting back outweigh the benefits, provided that the information is communicated appropriately.

Social science and environmental health researchers conducting studies involving CBPR, citizen science, biomonitoring, results report-back, and advocacy should take time to get to know the specific institutional culture of the IRB that will review their projects. Members within the same IRB may come from disparate disciplinary and community backgrounds and have varying ideas about the nature of research, especially CBPR and projects involving results report-back. IRB members may require education about the complex ethics and potential benefits of these genres of research that may appear to counter the usual clinical approaches to research ethics. Standard clinical approaches may not work well in some social or global contexts and may fail to protect or engage participants.

Familiarizing IRBs about the diversity of indigenous communities, research approaches, and ethical considerations can broaden traditional linear paradigms and biomedical understandings of ethics, which assert that individuals need to know their test results only when they present a known health risk. Protecting individual participants in research is important, but so too is protecting the long-term health, welfare, and autonomy of indigenous communities. IRBs need to be encouraged to consider the harms and benefits of research that remains undone. In the case of the BMPS, communities not given the opportunity to begin exploring the contaminant body burdens of mothers and their infants may make it more difficult for current and future generations to receive appropriate health care, including screening, diagnosis, and treatment for diseases that are known to be or potentially linked to toxic exposures. These communities lose opportunities to advocate for prevention such as working to ban harmful chemicals.

#### Differing ideas about research as advocacy

The NIEHS defines community-based participatory research (CBPR) as “a methodology that promotes active community involvement in the processes that shape research and intervention strategies, as well as in the conduct of research studies” [41]. As Minkler et al. [42, 43] note: “CBPR begins with a research topic of importance to the community with the aim of combining knowledge and action for social change to improve community health and eliminate health disparities.” Researchers and communities are encouraged to collaborate with one another for the purposes of mutual education. Researchers learn to value local and elder knowledge and to be sensitive to cultural differences and traditions, and community members learn how to collect and analyze data while infusing the research process with their own values, theories, and methods of inquiry.

However, not all researchers or IRB officials and committee members are familiar or accepting of CBPR. According to a former IRB member, the AAIRB committee members considered ACAT’s blend of research and advocacy problematic:

I think…having…the community that is…being investigated…be part of the process of creating the questions, how the questions are going to be answered, what the questions are makes a lot of sense. But…you need to have a line…you need to have a distinction between those, the people that are a member of the community and the people that are a member of the research team, and in some of these studies, they are one and the same. [Personal communication].

The AAIRB’s prejudices conflict with many of CBPR’s core principles, as well as a number of model practices that have been developed in conducting research with American Indian and environmental justice communities. Blurring the lines between researchers and participating communities can strengthen the rigor of research. Both groups can become more accountable to one another, and the answers to those questions have greater potential to be transformed into meaningful changes at the individual and societal levels.

The fact that the AAIRB’s definition of “community” excluded ACAT’s indigenous researchers and team members became a point of tension and conflict. The “community” representatives on the clinical advisory board for the AAIRB are volunteers from a variety of Alaska Native communities, who may or may not have the interests of other indigenous communities in mind while reviewing projects throughout their region. During an interview with Saxton, one clinician familiar with ACAT’s work expressed support of the BMPS. For decades, she observed firsthand some of the health disparities endured by St. Lawrence Island residents, such as high rates of cancer and miscarriages. She indicated that while great effort is made on the part of clinic advisory boards and the AAIRB to secure Alaska Native representation, it is not always clear that those volunteers have the affected community’s interests at stake. This ties in to long-standing differences and inequalities among the tribes in Alaska; some Alaska Native communities have vested interests in defending polluting industries that pay Native groups for extraction rights, while others protest these relationships because they conflict with concerns about community health. This demonstrates some of the potential problems during ethics reviews [3] without necessarily negating the importance of having indigenous representation on IRBs and clinical advisory boards [8].

#### Questioning the roles of IRBs and consequences of ethical imperialism

An IRB’s responsibility is to ensure that research with human participants incorporates the tenets of the Belmont Report: respect for persons, beneficence, and justice [44]. This entails assessing the protocol’s informed consent, risks and benefits, participant recruitment and selection procedures, and research methods. In the case of American Indian and Alaska Native research, the desires of Native communities and how they will benefit from research must be prioritized. IRBs can advise researchers on how to modify their projects to balance the risks and benefits, evaluate the qualifications of the principal investigators and ensure that proposed projects are within the researcher’s realm of expertise, and ensure that there are no conflicts of interest [45]. IRBs can deny approval to projects in which the risks—individual and or community level—outweigh the potential benefits.

The BMPS received support from the NIEHS and the Passport Foundation, both of which have high ethical standards and rigorous processes for evaluating researchers and project proposals. The AAIRB’s first review of the BMPS questioned the “scientific value” of the project, overstepping its mandate and engaging in research paternalism that would inhibit Native communities from exploring questions that are important to them. This leaves health concerns about toxics unresolved for present and future generations, thus raising other ethical concerns that were not considered by the AAIRB.

In repeatedly rejecting the BMPS proposal, the AAIRB precluded potential participants from the right to know what is in their bodies and what toxic legacies they may be passing on to future generations. The right to know the results of a biomonitoring project are vital to supporting the Belmont Report’s [44] core tenets of autonomy*,* beneficence, non-malfeasance, and justice [46, 47]. It is also in line with the indigenous values of autonomy and self-determination [48].

Since 2000, ACAT and the St. Lawrence Island communities have urged the military to clean up the PCBs completely from the formerly used defense sites at Northeast Cape and Gambell. ACAT also works at the state, national, and international levels for protective chemicals policies. The AAIRB, in blocking ACAT from conducting the BMPS, is inhibiting long-standing international efforts to collect data that could support meaningful and protective toxics policy changes that will have a long-lasting cumulative and beneficial impact on the health of Alaska Natives.

The repeated AAIRB rejections may also have caused communities to lose trust in research institutions, a key consequence given the historical legacy of research abuse and mistreatment of American Indian and Alaska Natives. These tensions may also discourage researchers from pursuing CBPR and citizen science projects that build capacity and empower communities. The struggle to gain IRB approval took time away from other important work and lowered the morale of ACAT researchers, especially the five Alaska Native research team members.

At the global level, ACAT had hoped to fill a gap in the WHO global breast milk biomonitoring program, which includes little data from the Arctic. Indigenous representatives for the Arctic need these data to support their work on the U.N. Stockholm Convention on Persistent Organic Pollutants, which promotes the precautionary principle to ban toxic chemicals. Indigenous Arctic environments and people, while far removed from industrial production and emissions, have some of the highest body burdens of toxic chemicals on the planet [49]. The AAIRB was reactive rather than reflective in its approach to the complex and multi-tiered ethics that would support such larger benefits.

## Conclusion

Although this case study focuses on Alaska Natives, the lessons are applicable to other groups, and should serve to educate IRBs broadly about the importance of local cultural context and of the value of full report-back of results to participants. In telling this story, our intention is not to cast blame; rather, we seek to help prepare researchers and communities as they confront these challenges during ethical reviews of their proposed studies. We encourage researchers who have conflicts with IRBs to share their experiences with others and to work collaboratively to develop legal, educational, and institutional strategies that counter research paternalism within IRBs, state and federal agencies, and corporations. The goal is to create reflective and process-oriented research ethics that are in tune with the realities and needs of the communities that partner with us.

## Abbreviations

AAIRB: Alaska Area Institutional Review Board
ACAT: Alaska Community Action on Toxics
BMPS: Breast Milk Pilot Study
CAB: Community Advisory Board
CBPR: Community Based Participatory Research
FUDS: Formerly Used Defense Sites
IHC: Indian Health Center
IHS: Indian Health Service
IRB: Institutional Review Board
MOMS: Making Our Milk Safe or MaPP
NIEHS: National Institute of Environmental Health Sciences
NSHC: Norton Sound Health Corporation
NSHC RERB: NSHC Ethics Review Board
OC: Organochlorine
PCB: Polychlorinated Biphenyl
POP: Persistent Organic Pollutant
