# Erratum to: Environmental health and justice and the right to research: institutional review board denials of community-based chemical biomonitoring of breast milk

**DOI:** 10.1186/s12940-016-0165-5

**Published:** 2016-07-29

**Authors:** Dvera I. Saxton, Phil Brown, Samarys Seguinot-Medina, Lorraine Eckstein, David O. Carpenter, Pamela Miller, Vi Waghiyi

**Affiliations:** 1 Department of Anthropology, College of Social Sciences, California State University, Fresno, 5242N. Backer Ave. Peters Business Building M/S 20, Fresno, CA 93740 USA; 2 Social Science Environmental Health Research Institute, Northeastern University, 318 INV, Boston, MA 02115 USA; 3 Alaska Community Action on Toxics, 505W. Northern Lights; Suite 205, Anchorage, AK 99503 USA; 4 Institute for Health and the Environment, University at Albany, 5 University Pl., Rm. A217, Rensselaer, NY 12144 USA; 5 Native Village of Savoonga Tribal Member, St. Lawrence Island, AK USA

## Erratum

Unfortunately, the original version of this article [1] did not include in the Acknowledgements section the complete funding information. The following sentence was missing: “This research was made possible in part the following grant “Ethical and Legal Challenges in Communicating Individual Biomonitoring and Personal Exposure Results to Study Participants” from the National Institute of Environmental Health Sciences #1R01ES017514-01A1”.

The corrected Acknowledgements have been included in full in this erratum.

## Acknowledgements

Partial support for this work comes from National Science Foundation grant GEO-1032754 (“Northeast Ethics Education Partnership for Research Ethics/Cultural Competence Training”) to Phil Brown. The authors wish to thank the peer reviewers and the editors of EH for their thoughtful comments and suggestions. The Breast Milk Pilot Study referred to in this article was funded by a National Institutes of Environmental Health five-year grant entitled “Environmental Health and Justice in Norton Sound, Alaska” and by an award from the Passport Foundation. All errors and omissions are our own. We are grateful to Susan Pinney for comments on the article. This research was made possible in part the following grant “Ethical and Legal Challenges in Communicating Individual Biomonitoring and Personal Exposure Results to Study Participants” from the National Institute of Environmental Health Sciences #1R01ES017514-01A1.
